# Supplementary material for: Meta-Analysis and Gene Set Enrichment Relative to ER Status Reveal Elevated Activity of MYC and E2F in the “Basal” Breast Cancer Subgroup
Source: PLoS One. 2009 Mar 9;4(3):e4710. doi: 10.1371/journal.pone.0004710 (PMC2650420; doi:10.1371/journal.pone.0004710)
Supplement: Dataset S1 — GSEA results from first screen. The file contains a zipped archive of the HTML results of the second GSEA screen. Reports for enriched or depleted datasets may be accessed from the “index.html” link within the my_analysis.GseaPreranked.1219975950625.rpt.zip ZIP archive. Enrichment plots are available for each of the 50 most enriched and depleted gene sets. (13.32 MB ZIP) [file pone.0004710.s001.zip › my_analysis.GseaPreranked.1219975950625.rpt.zip!//CHR5Q22.html]

Details for gene set CHR5Q22[GSEA]

|  || Dataset | zSco.ERneg.to.ERpos\_collapsed |
| Phenotype | NoPhenotypeAvailable |
| Upregulated in class | na\_neg |
| GeneSet | CHR5Q22 |
| Enrichment Score (ES) | -0.6585957 |
| Normalized Enrichment Score (NES) | -1.9683083 |
| Nominal p-value | 0.0024390244 |
| FDR q-value | 0.010534635 |
| FWER p-Value | 0.252 |
Table: GSEA Results Summary

  

Fig 1: Enrichment plot: CHR5Q22      
 Profile of the Running ES Score & Positions of GeneSet Members on the Rank Ordered List

  

| PROBE | GENE SYMBOL | GENE\_TITLE | RANK IN GENE LIST | RANK METRIC SCORE | RUNNING ES | CORE ENRICHMENT || 1 | CD14 |  |  | 1768 | 2.716 | -0.0595 | No |
| 2 | AP3S1 |  |  | 4166 | 1.445 | -0.2005 | No |
| 3 | C5ORF13 |  |  | 6512 | 0.645 | -0.3593 | No |
| 4 | TSSK1 |  |  | 7023 | 0.479 | -0.3847 | No |
| 5 | U2AF1L1 |  |  | 9522 | -0.572 | -0.5570 | No |
| 6 | YTHDC2 |  |  | 10875 | -1.446 | -0.6195 | Yes |
| 7 | TRIM36 |  |  | 10925 | -1.503 | -0.5826 | Yes |
| 8 | FLJ11235 |  |  | 11452 | -1.991 | -0.5683 | Yes |
| 9 | ISOC1 |  |  | 11714 | -2.233 | -0.5275 | Yes |
| 10 | TMED7 |  |  | 12091 | -2.698 | -0.4828 | Yes |
| 11 | DCP2 |  |  | 12194 | -2.836 | -0.4138 | Yes |
| 12 | FEM1C |  |  | 12384 | -3.083 | -0.3447 | Yes |
| 13 | PJA2 |  |  | 13093 | -4.800 | -0.2681 | Yes |
| 14 | REEP5 |  |  | 13138 | -4.972 | -0.1370 | Yes |
| 15 | DMXL1 |  |  | 13218 | -5.571 | 0.0077 | Yes |
Table: GSEA details [plain text format]

  

Fig 2: CHR5Q22: Random ES distribution      
 Gene set null distribution of ES for **CHR5Q22**
